# Establishing hospital-specific background microbial libraries to reduce false positives in mNGS diagnosis of periprosthetic joint infection

**DOI:** 10.3389/fcimb.2025.1668697

**Published:** 2026-01-26

**Authors:** Yinguang Cao, Chengtan Wang, Han Yin, Duliang Xu, Wei Li, Zhenfeng Yuan, Wenbin Xu, Zhenzhu Song, Feng Pang, Dawei Wang

**Affiliations:** 1 Department of Clinical Laboratory, Liaocheng Key Laboratory of Medical Microbiology and Immunology, Liaocheng People’s Hospital, Liaocheng, Shandong, China; 2 National Health Commission Key Laboratory of Systems Biology of Pathogens, State Key Laboratory of Respiratory Health and Multimorbidity and Christophe Mérieux Laboratory, National Institute of Pathogen Biology, Chinese Academy of Medical Sciences and Peking Union Medical College, Beijing, China; 3 Department of Orthopaedics, Beijing Jishuitan Hospital Liaocheng Hospital, Shandong, China; 4 Department of Orthopaedics, Liaocheng People’s Hospital, Liaocheng, Shandong, China; 5 Department of Clinical Laboratory, Beijing Jishuitan Hospital Liaocheng Hospital, Liaocheng, Shandong, China

**Keywords:** arthroplasty, bacterial culture, next-generation sequencing, joint replacement, periprosthetic joint infection, background microbial libraries

## Abstract

**Background:**

Due to the high sensitivity of metagenomic next-generation sequencing (mNGS), trace amounts of nucleic acid contamination can lead to false positives, posing challenges for result interpretation. This study is the first to experimentally identify and establish background microbial libraries (BML) related to periprosthetic joint infection (PJI) across different medical institutions, aiming to demonstrate the necessity of institution-specific BMLs to improve mNGS diagnostic accuracy.

**Methods:**

Samples were taken from 3 different acetabular reamer for hip arthroplasty in 7 different hospitals. The whole process was strictly aseptic, mNGS was performed according to standard operating procedures. The sterility of instruments was confirmed by culture method. The sequencing results of specimens from different hospitals were compared to analyze the difference of background bacteria. Bioinformatics analysis and visualization were presented through R language.

**Results:**

A total of 26 samples (24 instrument swabs and 2 negative controls) generated 254 million reads, of which 1.13% matched microbial genomes. The proportion of microbial reads (1.13%) falls within ranges typically observed for contamination in low-biomass metagenomic sequencing studies. Among these, bacteria accounted for 87.48%, fungi 11.18%, parasites 1.26%, and viruses 0.06%. The most abundant bacterial genera included Cutibacterium, Staphylococcus, and Acinetobacter. Principal component analysis revealed distinct bacterial compositions among the seven hospitals, and clustering analysis showed significant inter-hospital variation (*p* < 0.05). Liaocheng People’s Hospital exhibited the highest species richness (340 species), followed by Guanxian County People’s Hospital (169 species).

**Conclusions:**

The composition and abundance of residual bacterial DNA vary markedly among institutions, underscoring the necessity of establishing hospital-specific BMLs. Incorporating such libraries into clinical mNGS interpretation can effectively reduce false positives and enhance the diagnostic accuracy of PJI. arthroplasty, bacterial culture, next-generation sequencing, joint replacement, periprosthetic joint infection, background microbial libraries.

## Introduction

Periprosthetic joint infection (PJI) is one of the serious complications after total joint replacement (McNally et al., 2021). Timely and accurate microbiological diagnosis is crucial for effective infection control and clinical decision-making (Huang et al., 2020). False-negative results due to low activity or low concentrations of bacteria, and difficult-to-cultivate pathogens *in vitro* are common limitations of this traditional method (Berbari et al., 2007; Font-Vizcarra et al., 2010; Parvizi et al., 2014; Tan et al., 2018; Tarabichi et al., 2018a). The metagenomic next-generation sequencing (mNGS) is a molecular detection method for pathogens that have attracted great attention in recent years. This method detects all microbial nucleic acid fragments in a specimen by unbiased shotgun sequencing and prompts its correlation with clinical infection. The mNGS technology has demonstrated broad application in pathogen identification of PJI cases (Goswami and Parvizi, 2020; Huang et al., 2020; Huang et al., 2020; He et al., 2021). Through improving the sensitivity of pathogen detection, mNGS has a positive impact on improving the prognosis of PJI patients, rational use of antibiotics, and reducing the economic burden of patients (Wang et al., 2020).

Because of its extremely high analytical sensitivity, even trace amounts of exogenous nucleic acids introduced during sampling, reagent handling, or laboratory procedures can generate false-positive signals, which is one of the biggest challenges in interpreting mNGS testing reports. Street et al. concluded that high levels of DNA contamination may be introduced in the process of mNGS, which is difficult to remove and can only be minimized during the operation (Street et al., 2017). Huang et al. also reported the interference of exogenous DNA contamination in the detection of bone and joint infections using mNGS (Huang et al., 2019). Detecting contaminating nucleic acid sequences during mNGS and mistaking them for pathogenic microorganisms may be a common problem for all inspectors (Tarabichi et al., 2018b). Such misclassification directly affects clinical decisions, including unnecessary antimicrobial therapy, prolonged hospitalization, inappropriate revision surgery, and increased healthcare costs.

Given that contamination patterns vary by institution, workflow, and laboratory environment, the lack of standardized background microbial references increases the risk of false-positive interpretation in PJI diagnostics. Therefore, establishing institution-specific background microbial libraries (BMLs) is essential to correctly distinguish true pathogens from residual environmental or reagent-derived signals.

In this study, for the first time, we experimentally detected and established PJI-related interfering nucleic acid BML in different medical institutions. We obtained residual nucleic acid on the surface of instruments for joint arthroplastyin different medical institutions through mNGS, and applied bioinformatics methods to analyze and compare the differences in microbial distribution. To more accurately apply mNGS to diagnose PJI diseases, each medical institution must establish its BML database.

## Materials and methods

### Experimental design and sample acquisition

Samples were taken from 7 hospitals in Liaocheng City (Shandong Province, China), including Liaocheng People’s Hospital (No. LPH 1-3, and No. LPH 4–6 containing human Hela cells), Liaocheng Hospital of Traditional Chinese Medicine (No. LHTCM 1-3), Dong’e County People’s Hospital (No. DCPH 1-3), Guanxian County People’s Hospital (No. GCPH 1-3), Yanggu County People’s Hospital (No. YCPH 1-3), Shenxian County People’s Hospital (No. SCPH 1-3), Chaocheng County People’s Hospital (No. CCPH 1-3). All seven hospitals are located within the same province and share similar case mixes for joint arthroplasty, with no major differences in patient populations or surgical types that would account for the observed microbial variation. Three different acetabular reamer for hip arthroplasty were collected from each hospital. Among them, Liaocheng People’s Hospital took samples twice. In one experiment, HeLa cells were added to a subset of samples (LPH4–LPH6) to evaluate whether increased human DNA content affects the detection of background microbial sequences, while the corresponding samples from the same instrument set (LPH1–LPH3) were sequenced directly without HeLa addition. Samples from the other six hospitals were all processed and sequenced without the introduction of human cells.

All specimens were collected in a thousand-level laminar flow operating room that had constant temperature and humidity to minimize contamination. Sampling personnel are dressed in accordance with sterile protection requirements, with hats, masks, and gloves. Throughout the procedure, avoid talking to prevent possible dental bacterial contamination. Before aseptic surgery, take a special swab to wipe the surface of the acetabular reamer, and mix it with sterile water after wiping. The treated liquid was divided into two parts as sample treatment solution, one for microbial culture and the other for sequencing. In addition to the conventional internal reference and quality control substances in the sequencing process, this experiment set up a swab (Control 1) and ultrapure water (Control 2) without wiping any instruments involved as additional control substances. All controls follow the normal test procedure until the report is obtained.

### Sample size justification

This study was exploratory in nature and aimed to characterize background microbial profiles across different medical institutions rather than to test a predefined statistical hypothesis. Therefore, the sample size was determined by the number of participating hospitals and the availability of aseptically processed instruments. A total of 26 samples (including 24 instrument swabs and 2 negative controls) was sufficient to capture reproducible background microbial patterns, consistent with prior mNGS contamination-profiling studies that typically include 10–40 samples (Salter et al., 2014; Thoendel et al., 2018; Eisenhofer et al., 2019).

### Microbial culture

The sample treatment solution was inoculated in 0.1 ml aliquots on aerobic blood agar, anaerobic blood agar, and fungal Sabouraud agar. Then they were incubated under the corresponding temperature and environmental conditions, respectively, 35°C and CO2 content of 5-7% for 6 days aerobic culture, 14 days in the anaerobic tank for anaerobic culture, and 28°C incubators for fungus medium 28 days. Additionally, 1 ml of the treatment solution was injected into the BACTEC Peds Plus/F bottle and incubated for 6 days in the BACTEC FX200 automated system. Furthermore, mycobacterial cultures were performed in liquid cultures for 42 days by a BACTEC MGIT 960 system (Becton-Dickinson, USA). Any growth from the treatment fluid was considered positive.

### Sample processing and metagenomic sequencing

All experiments were performed in a certified clean molecular laboratory with physically separated workflow zones. DNA was extracted from instrument swabs using the TIANamp Micro DNA Kit (TIANGEN, China). Library construction followed the manufacturer’s standard protocol (MGI DNA construction kit), including enzymatic fragmentation, end-repair, adaptor ligation, and PCR amplification. Sequencing was performed on the BGISEQ-50 platform using single-end 50 bp reads with a target yield of 20 million reads per sample. Each batch included a positive control (Acinetobacter baumannii ATCC 19606) and two negative controls (unsampled swab and nuclease-free water), which underwent all experimental steps in parallel.

### Bioinformatic analysis

Low-quality reads and adaptors were removed, and human-derived sequences were filtered by mapping to the hg38 reference genome using BWA. Remaining reads were aligned to a curated microbial reference database derived from NCBI, containing bacterial, viral, fungal, parasite, and mycoplasma genomes. Taxonomic assignment and strict mapping read counts (SMRN) were generated for each microbial species and genus. Relative and absolute abundances, coverage, depth, and Shannon diversity indices were calculated. Complete details, including software parameters and reference database composition, are provided in the **Supplementary Methods**.

### Data aggregation and visualization

Bioinformatics analysis and visualization using the relative abundance of different microbial genera as indicators are all realized by R language. The principal component analysis uses the “PCA tools” R package; heatmaps use the “pheatmap” R package. Other analysis and data preprocessing use R language built-in functions or the “ggplot2” R package.

## Results

### Culture-based sterility verification of surgical instruments prior to mNGS analysis

All samples were negative for aerobic, anaerobic, fungal, and mycobacterial cultures throughout the designated incubation periods. No microbial growth was observed, including after enrichment in automated blood culture systems and extended incubation in mycobacterial culture conditions, confirming the absence of viable microorganisms on instrument surfaces under standard culture-based detection.

### Overview of sequencing output and taxonomic composition of residual nucleic acid on sterile surgical instruments

**Figure 1:**
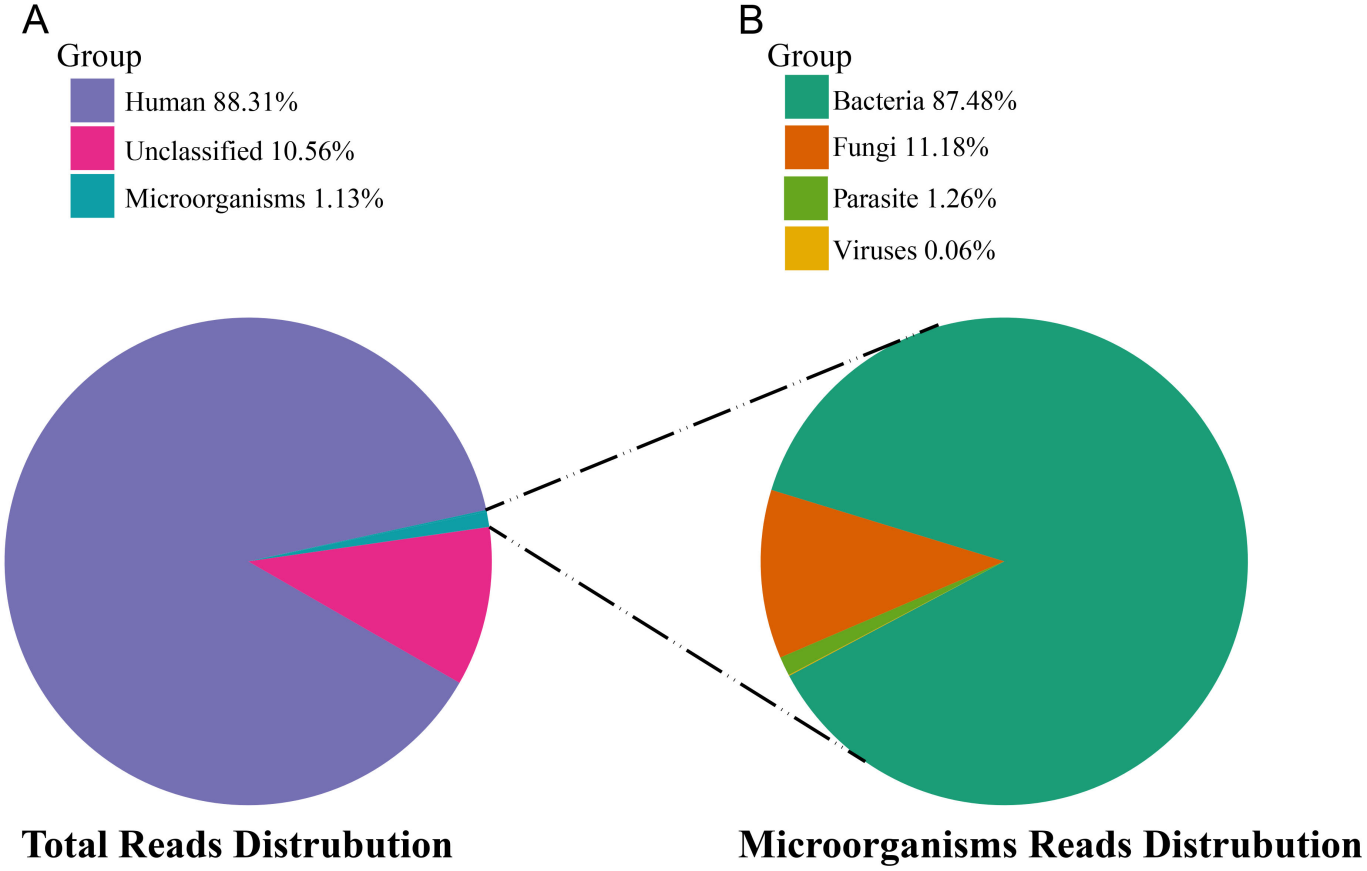
Taxonomic composition of mNGS reads from sterile instrument swab samples. **(A)** Reads distribution of total DNA in all environmental samples. **(B)** Distribution of microbial reads in the absence of human and unclassified reads.

A total of 26 samples from 7 different medical institutions were included in this study, including 24 instrument swab samples and 1 blank control (Control 1), and 1 blank water control (Control 2). In these 26 samples, 254,314,707 fragment reads were obtained by sequencing, of which 25,210,926 were internal reference sequences (detection rate of internal reference was 100%). The Clean Reads obtained after removing adapter sequences and low-quality sequencing data were aligned to the human reference genome, finally, 88.31% could be aligned to the human reference genome, 10.56% were not aligned to the specific genome, and 1.13% compared to microbial genomes (**Figure 1A**), of which bacterial sequences account for 87.48%, fungal sequences account for 11.18%, parasite sequences account for 1.27%, and virus sequences account for 0.06% (**Figure 1B**).

### Principal component analysis reveals institution-specific microbial DNA patterns and minor batch effects

**Figure 2:**
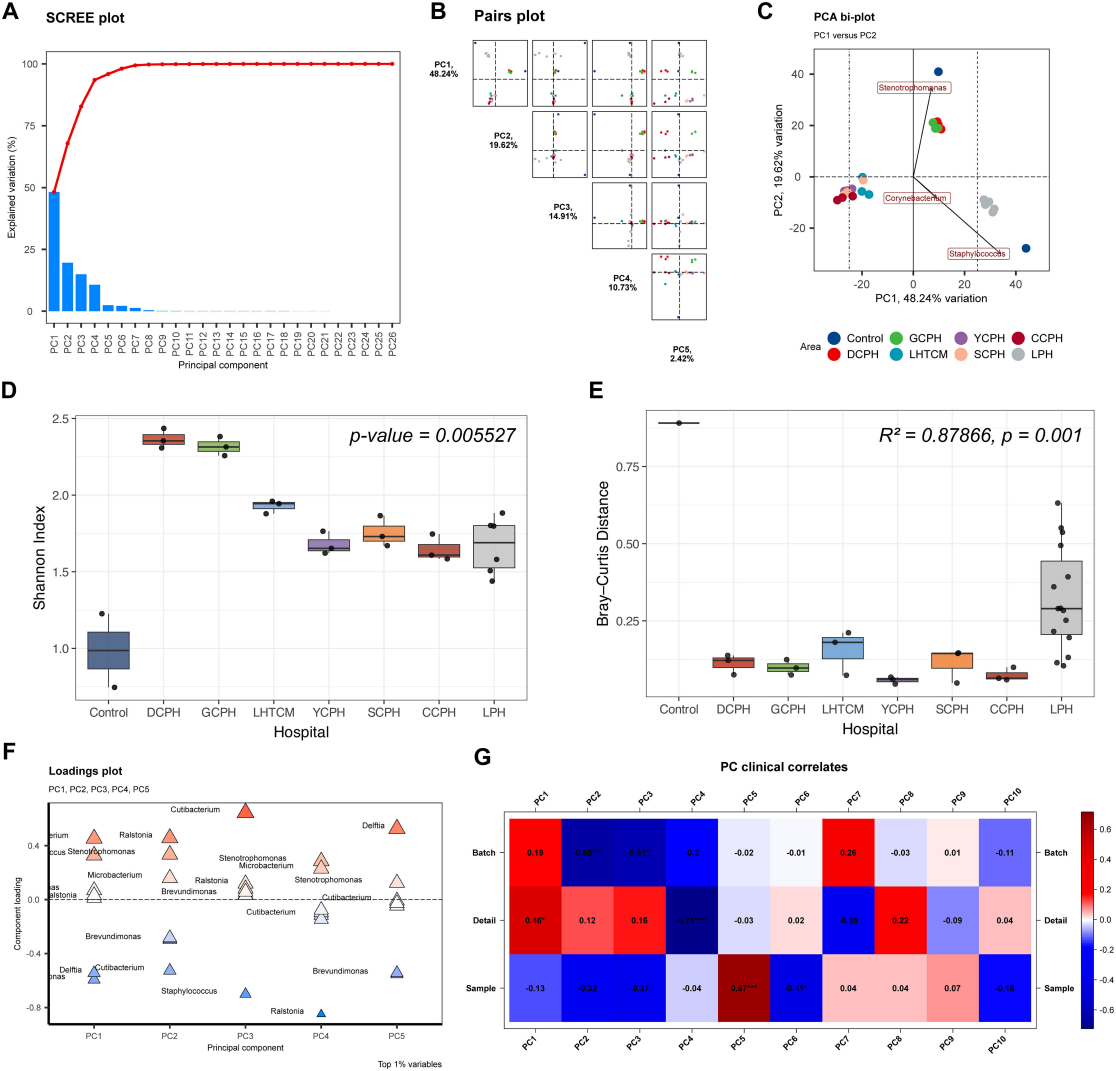
Principal Component Analysis of the microbiome composition. **(A)** Scree plot showing the proportion of variance explained by each principal component, used to determine the number of components retained. **(B)** Pairs plot illustrating sample dispersion and clustering across multiple principal component (PC) dimensions. **(C)** PCA biplot displaying sample distribution along PC1 and PC2, with loading vectors indicating the genera contributing most to variation between hospitals. **(D)** Shannon diversity index across hospitals, showing significant inter-hospital differences. **(E)** Within-hospital Bray–Curtis distances, indicating significant differences in community composition across hospitals. **(F)** Loading plot depicting the major microbial genera contributing to the first five principal components. **(G)** PC clinical correlates, showing the association between principal components and sample metadata (Batch, Detail, Sample).

We performed principal component analysis (PCA) to characterize variation in background microbial DNA profiles across the seven participating medical institutions. The scree plot (**Figure 2A**) indicated that the first several principal components captured the majority of variance. Pairwise PC projections (**Figure 2B**) and the PCA biplot (**Figure 2C**) showed clear sample clustering by hospital, demonstrating distinct institution-specific microbial DNA signatures on instrument surfaces. PC1 (48.24%) and PC2 (19.62%) together explained 67.86% of the variance, indicating that most of the observed differences in background microbial composition were driven by hospital-specific factors. Shannon diversity analysis (**Figure 2D**) revealed significant differences in alpha diversity among hospitals. Similarly, within-hospital Bray–Curtis distance comparisons (**Figure 2E**) showed marked variability between institutions, with PERMANOVA confirming highly significant inter-hospital differences in community structure. The loading plot (**Figure 2F**) identified the genera contributing most strongly to principal component separation, and the PCA–metadata correlation map (**Figure 2G**) indicated that hospital identity was the primary driver of microbial variation, with only minor batch effects. Collectively, these results demonstrate that background microbial DNA patterns are highly institution-specific, reinforcing the necessity of establishing independent background microbial libraries for accurate mNGS interpretation. To further summarize these inter-hospital differences, we aggregated genus-level abundance data and identified the top 15 genera for each medical institution. The resulting dominant-genus profiles are provided in **Supplementary File 1**, offering a clear overview of the taxa most characteristic of each hospital.

### Inter-hospital comparison of bacterial diversity reveals distinct background signatures on instrument surfaces

**Figure 3:**
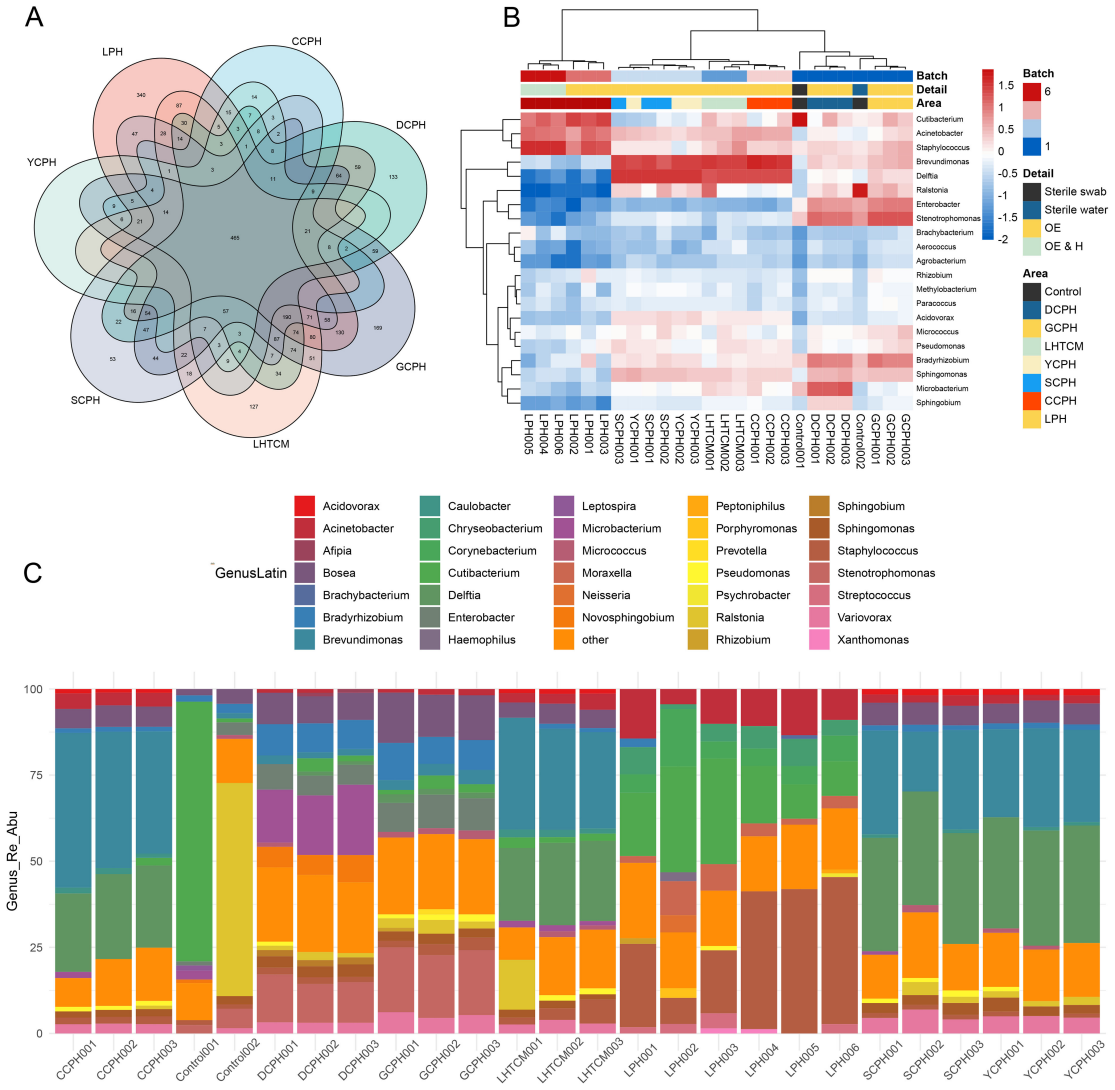
The distribution of bacteria on the surfaces of instruments varied between hospitals, as did the characteristic flora of each healthcare facility **(A)** Venn diagram, show the common and unique types of bacteria among different medical institutions. **(B)** Heat map of the relative abundance at the genus level of bacteria, Demonstrates similarities and differences in the distribution of bacterial genus levels in different medical institutions. **(C)** Cumulative map of the relative abundance of bacterial spp. levels in different healthcare facilities, demonstrates the distribution of the dominant genera in different healthcare facilities.

To characterize the shared and unique bacterial communities across institutions, Venn diagram analysis identified a total of 465 bacterial species, with Liaocheng People’s Hospital showing the greatest diversity (340 species), followed by Guanxian County People’s Hospital (169 species) (**Figure 3A**). Hierarchical clustering of genus-level relative abundance demonstrated high intra-hospital consistency and marked inter-hospital variation (**Figure 3B**). Notably, samples with added exogenous human DNA (HeLa cells; LPH4–6) exhibited microbial profiles similar to their unmodified counterparts (LPH1–3), indicating negligible impact from human nucleic acid contamination. Common background genera, including Cutibacterium, Acinetobacter, and Staphylococcus, were found across most sites; however, the dominant taxa and their abundances varied significantly among institutions (**Figure 3C**), suggesting hospital-specific contamination patterns likely driven by localized environmental or historical factors.

### Analysis of non-bacterial microbial communities: fungal, viral, and parasitic DNA profiles

To further characterize the non-bacterial components of residual microbial DNA, we analyzed fungal, viral, and parasitic communities across all samples using **Supplementary Figures S1**-**S6**. PCA of fungal reads revealed scattered clustering patterns with no institution-specific grouping, indicating low and inconsistent fungal abundance across hospitals (**Supplementary Figure 1**). The genus-level heatmap and relative abundance plot (**Supplementary Figure 2**) confirmed the presence of diverse fungal taxa such as Candida, Aspergillus, and Trichoderma, although at low overall abundance. Viral community analysis (**Supplementary Figures 3**, **4**) identified limited reads, primarily from human papillomaviruses, adenoviruses, and polyomaviruses, with no dominant species or regional pattern. Similarly, parasitic reads (**Supplementary Figures 5**, **6**) were sparse and taxonomically diverse, including genera such as Plasmodium, Trichinella, and Acanthamoeba, again lacking consistent distribution across institutions. These findings suggest that, in contrast to the bacterial component, fungal, viral, and parasitic DNA contributed minimally to the overall microbial background and exhibited no clear institutional specificity.

## Discussion

At present, although a small number of studies have focused on the contamination of background nucleic acid fragments in mNGS sequencing, most of them are limited to empirical speculation (Thoendel et al., 2018). In this study, we demonstrated that residual nucleic acid fragments persist on acetabular reamers used for joint arthroplasty across different medical institutions, despite complete inactivation of viable microorganisms by routine sterilization. These background DNA signals, predominantly bacterial in origin, varied markedly between hospitals and formed institution-specific microbial patterns. Such residual fragments are not removed by standard disinfection because DNA is more stable than viable organisms, and their presence can lead to false-positive pathogen detection in mNGS. Given that mNGS reports often guide antimicrobial selection and surgical planning in PJI, misinterpreting background DNA as true infection poses a risk of unnecessary antibiotic exposure or inappropriate operative intervention. Our findings therefore highlight the clinical importance of recognizing and accounting for institution-specific background microbial DNA when interpreting mNGS results for PJI diagnosis.

Recent studies on PJI etiology have shown that Staphylococcus remains the most common pathogen, with gram-positive cocci, gram-negative bacilli, anaerobes, and Candida also contributing to infection, while rare organisms such as Aspergillus fumigatus, Actinomyces, and Mycoplasma hominis have been occasionally reported (Del Pozo and Patel, 2009; Cobo and Del Pozo, 2011; Benito et al., 2016; Bassetti et al., 2019). Given this microbial complexity, mNGS provides important diagnostic value, but its extremely high sensitivity makes it susceptible to false-positive signals generated by trace nucleic acid residues introduced during sampling or laboratory handling. Although sterile, nucleic-acid–free consumables are used, residual DNA in reagents or the instrument environment can still interfere with interpretation. In this study, two synchronized negative controls confirmed that detection reagents did not influence inter-hospital microbial differences. However, the presence of stable background DNA fragments on surgical instruments remains a critical challenge, particularly because many of the interfering taxa detected here (e.g., Corynebacterium, Novosphingobium, Sphingomonas) are uncommon in PJI yet may occasionally cause infection in immunocompromised patients (Street et al., 2017; Palan et al., 2019; Huang et al., 2020; Indelli et al., 2021). More importantly, some residues overlapped with common PJI pathogens such as Staphylococcus and Acinetobacter, complicating clinical interpretation.

Previous work by Thoendel et al. demonstrated that contaminating microbial DNA in metagenomic workflows, especially from reagents, complicates interpretation of PJI sonicate fluid sequencing (Thoendel et al., 2018). Their study identified characteristic reagent-derived contaminants such as Acinetobacter, Streptococcus, Cutibacterium, and Staphylococcus and highlighted the challenge of distinguishing these organisms from true pathogens. Our findings expand upon this by showing that contamination is not limited to reagents: even after complete sterilization, surgical instruments retain institution-specific microbial DNA signatures that vary substantially across hospitals. This suggests that, in addition to reagent contamination described by Thoendel et al., localized environmental or instrument-derived nucleic acids represent an additional and previously underrecognized source of background noise. By characterizing these hospital-level background profiles, our dataset complements and extends prior work, providing practical evidence supporting the need for institution-specific BMLs to improve the accuracy of mNGS-based PJI diagnosis.

Establishing institution-specific BMLs therefore provides an essential reference for interpreting mNGS results, although some contaminant taxa may still be difficult to distinguish from true pathogens. Determining the clinical significance of detected organisms requires integrated assessment using bioinformatic features (such as coverage and abundance), reagent background monitoring, and clinical context. For pathogens that are part of the human microbiome—such as coagulase-negative Staphylococci or Dermatobacter acnes—even quantitative thresholds may not reliably separate contamination from infection (Tande and Patel, 2014; Davidson et al., 2019; Rao et al., 2020). Consequently, mNGS interpretation must incorporate multiple factors, including patient condition, joint type, geographic variability, and timing relative to surgery, particularly in low-abundance infections where contamination can obscure true pathogen signals.

Furthermore, although bacteria constituted the majority of background microbial reads, low-level fungal, viral, and parasitic sequences were also detected across institutions. These non-bacterial signals are clinically relevant because mNGS reports frequently include rare fungal or viral reads that may be misinterpreted as true infections, often originating from bioinformatic artifacts (such as the misclassification of host DNA as Sporozoa) (Pain et al., 2008; Gu et al., 2019) or trace reagent contamination (the “kitome”) (Salter et al, 2014). Establishing hospital-specific background profiles for these microbial groups helps distinguish sporadic environmental or reagent-derived nucleic acids from clinically meaningful pathogens, thereby reducing unnecessary antifungal or antiviral treatments and improving the specificity of mNGS-based PJI diagnosis.

In addition to characterizing background microbial profiles, several practical strategies may help mitigate the influence of environmental or reagent-derived contamination in clinical mNGS workflows. Routine inclusion of reagent blanks, periodic environmental monitoring, and the application of statistical filtering approaches (such as abundance-based thresholds or contaminant-identification algorithms) can assist in identifying low-level background signals. Institution-specific BMLs can be further integrated into this framework by periodically sequencing sterilized instruments, negative controls, or operating-room environmental samples and storing the resulting background profiles in a local reference repository. These BMLs may be updated on a scheduled basis—such as every 3–6 months—or whenever laboratory workflows, reagent batches, or disinfection procedures undergo major changes. During routine mNGS interpretation, organisms detected in clinical specimens can then be compared against the institution’s BML to determine whether they reflect likely contaminants or true pathogens. Such combined mitigation and implementation strategies may substantially improve the accuracy and clinical utility of mNGS-based PJI diagnostics.

In interpreting our findings, it is essential to recognize that metagenomic sequencing data from low-biomass samples must be evaluated with caution and within the appropriate clinical context. The extremely high analytical sensitivity of mNGS allows trace environmental or reagent-derived nucleic acids to be detected alongside true pathogens, and such signals do not inherently imply clinical infection. In this study, low-abundance protozoan and viral reads were identified, but these organisms are biologically implausible causes of PJI and were therefore regarded as background metagenomic noise rather than meaningful microbiological findings. These observations underscore that mNGS should not be used as a stand-alone diagnostic tool. Accurate interpretation requires integration of sequencing results with clinical presentation, imaging, inflammatory markers, intraoperative findings, and conventional microbiology. Effective dialogue among clinicians, microbiologists, and bioinformaticians is critical to avoid overinterpretation of sequencing artefacts and to determine whether detected organisms have true pathogenic relevance. Our findings highlight that institution-specific background microbial libraries serve not as independent diagnostic criteria but as contextual references that support multidisciplinary evaluation and help distinguish genuine pathogens from environmental or reagent-derived signals during clinical decision-making.

The study still has several limitations. First, the sample size per institution was relatively small, which limits the statistical power of this exploratory analysis. Although clear inter-hospital clustering patterns and significant diversity differences were observed, larger and longitudinal sampling will be needed to further validate the robustness of these findings. Second, although we collected samples from multiple hospitals during the same period, we did not perform continuous monitoring of background microbial profiles across different time intervals. Similar to routine environmental microbiological surveillance in medical institutions based on culture-based assessments, periodic sequencing of residual nucleic acids from instruments used in joint arthroplasty may be required in the future. Third, because the sequences of residual viral and fungal fragments were very low, false-positive interference during formal diagnostic testing cannot be completely excluded. Fungal nucleic acids, especially from filamentous fungi, are difficult to extract due to their thick cell walls; therefore, interpretation rules for fungi cannot be directly applied using bacterial thresholds.

## Conclusion

The surface of the instrument for joint replacement sterilized by the routine process does not contain any active microorganisms, but there are residual microbial nucleic acid fragments, which will interfere with the mNGS test results. The residual microbial nucleic acid fragments on the surface of joint replacement devices are mainly bacterial DNA, and there are differences in the distribution of bacteria between different medical institutions, which may be related to the historical cases handled by the medical treatment. In the current form, it is necessary to establish independent BML in different medical institutions to improve the accuracy of mNGS on the diagnosis of PJI.

## Data Availability

The datasets supporting the conclusions of this article are not available in an open access repository because the authors have not finished the data analysis yet. If anyone is interested in exploring specific issue, please contact Prof. Dawei Wang.
