# Supplementary material for: Establishing hospital-specific background microbial libraries to reduce false positives in mNGS diagnosis of periprosthetic joint infection
Source: Front Cell Infect Microbiol. 2026 Jan 26;15:1668697. doi: 10.3389/fcimb.2025.1668697 (PMC12883815; doi:10.3389/fcimb.2025.1668697)
Supplement: Supplementary file 2 [file DataSheet2.docx]

**Supplementary Figures**

**Supplementary Figure 1. Principal component analysis of the distribution of fungi communities.**

(A) Scree plot, determine the number of principal components to keep in a principal component analysis. (B) Pairs plot demonstrates the dispersion and aggregation of samples at different PC scales. (C) PCA bi plot demonstrates the dispersion and aggregation of samples at two main PC scales. (D) Loadings plot, determine the variables that drive variation among each PC. (E) PC clinical correlates, Correlate the principal components back to the clinical data (Batch, Detail, Sample).

**Supplementary Figure 2. Genus-Level Composition and Inter-Hospital Variation of Fungal Communities**

Top: Heatmap showing the relative abundance of fungal genera across all samples, highlighting intra- and inter-hospital differences. Sample metadata (batch, swab type, and institution) are annotated above.

Bottom: Stacked bar plot of the dominant fungal genera, illustrating the compositional diversity and relative abundance in each sample from different medical institutions.

**Supplementary Figure 3. Principal component analysis of the distribution of virus communities.**

(A) Scree plot, determine the number of principal components to keep in a principal component analysis. (B) Pairs plot demonstrates the dispersion and aggregation of samples at different PC scales. (C) PCA bi plot demonstrates the dispersion and aggregation of samples at two main PC scales. (D) Loadings plot, determine the variables that drive variation among each PC. (E) PC clinical correlates, Correlate the principal components back to the clinical data (Batch, Detail, Sample).

**Supplementary Figure 4. Species-Level Characterization of Viral Communities Across Medical Institutions**

Top: Heatmap of viral species relative abundance, showing compositional similarities and differences among samples from various hospitals. Sample metadata including batch, swab type, and institution are annotated above.

Bottom: Stacked bar chart illustrating the relative abundance of dominant viral species in each sample, highlighting the variability in viral composition between institution.

**Supplementary Figure 5. Principal component analysis of the distribution of parasite communities.**

(A) Scree plot, determine the number of principal components to keep in a principal component analysis. (B) Pairs plot demonstrates the dispersion and aggregation of samples at different PC scales. (C) PCA bi plot, demonstrates the dispersion and aggregation of samples at two main PC scales. (D) Loadings plot, determine the variables that drive variation among each PC. (E) PC clinical correlates, Correlate the principal components back to the clinical data (Batch, Detail, Sample).

**Supplementary Figure 6. Species-Level Profiling of Parasitic DNA Reveals Low-Abundance and Diverse Signatures Across Institutions**

Top: Heatmap showing the relative abundance of parasitic species detected in instrument swab samples from eight medical institutions. Metadata annotations indicate batch, sampling detail, and hospital source.

Bottom: Stacked bar plot illustrating the composition and distribution of dominant parasitic species in each sample, highlighting high diversity but generally low abundance across sites.
