# Supplementary material for: Establishing hospital-specific background microbial libraries to reduce false positives in mNGS diagnosis of periprosthetic joint infection
Source: Front Cell Infect Microbiol. 2026 Jan 26;15:1668697. doi: 10.3389/fcimb.2025.1668697 (PMC12883815; doi:10.3389/fcimb.2025.1668697)
Supplement: Supplementary file 3 [file DataSheet3.docx]

**Supplementary Methods**

**Sample Preparation**

All steps are performed in a standard four independent zone design dedicated gene amplification laboratory authorized by the management department. The laboratory is equipped with a laminar flow device to ensure the requirements of airflow and cleanliness. Pre-processing of samples and DNA extraction were performed in a Class B2 safety cabinet. The routine quality control used the internal standard provided by the manufacturer (MGI Tech Co., Ltd, Wuhan, China), and 105 CFU of *Acinetobacter baumannii* ATCC 19606 suspended in Ringer's solution was used as a positive external control. Pure water without added template DNA was used as a negative control. Negative and positive controls follow all experimental procedures. This process does not introduce a microbial DNA enrichment step. According to the standard procedure of DNA sequencing provided by BGI Genomics, a wall-breaking reagent (MGI Tech Co., Ltd, Wuhan, China) containing a wall-lysing enzyme was added for the wall-breaking treatment of magnetic beads.

**DNA Extraction and Purification**

DNA was extracted using the column extraction kit TIANamp Micro DNA Kit (DP316, TIANGEN Biotech, Jiangsu, China). The concentration of the extracted nucleic acid was determined by the Qubit dsDNA HS Assay kit using Qubit Fluorometer 4.0 (Invitrogen, Singapore). Nucleic acid fragmentation was performed by an enzyme digestion kit (MGI Tech Co., Ltd, Wuhan, China), and the gDNA was cut into fragments of about 250bp-350bp. Purification and DNA elution in TE pH 8.0 were performed with factory-supplied magnetic beads (MGI Tech Co., Ltd, Wuhan, China).

**Next-Generation Sequencing**

DNA libraries were prepared using the MGI DNA construction kit (MGI Tech Co., Ltd, Wuhan, China) after end-repair, adapter-ligation, and PCR amplification. Library construction and Pooling were performed in strict accordance with the instructions. The input amount of nucleic acid for single library construction is 1-100 ng, and the number of PCR cycles is 16. Use nucleic acid-free water as the library eluate. The constructed library was qualified by ExKubit dsDNA Assay Kit by Agilent 2100 (Agilent Technologies, Santa Clara, CA) and Qubit 4.0 (Invitrogen) for quality control. The library concentration quality control standard was double-stranded DNA concentration >1.0 ng/μl, fragment size ~280 bp. Convert to a single-stranded circular DNA library by DNA denaturation and circularization. DNA Nanospheres (DNB) were generated from single-stranded circular DNA using rolling circle amplification (RCA), and DNB preparation was performed according to a protocol provided by BGI Genomics. The concentration of DNB was determined with the Invitrogen Qubit ssDNA Assay Kit using Qubit 4.0, and the range of 8-40ng/μL is qualified. According to the combined probe-anchored polymerization sequencing method, the Pmseq TM infection pathogen high-throughput detection kit (MGI Tech Co., Ltd, Wuhan, China) was used to load DNB into the sequencing reaction universal kit (MGI Tech Co., Ltd, Wuhan, China), and sequenced on the BGISEQ-50 platform (BGI Genomics, Wuhan, China). The reads sequencing strategy is single-end, the read length is 50 (nt), the index sequencing strategy is double-ending, and the index read length is 10 (nt). The single-sample data yield standard is 20M reads.

**Metagenomic Data Analysis**

First, the raw data of sequencing is processed, and high-quality data is obtained after removing adapter sequences and low-quality sequences. Then the high-quality data from sequencing were mapped to the human genome (hg19) using Burrows-Wheeler Alignment (BWA)[1], annotated and striped human genome data. The remaining sequencing data were simultaneously aligned to 4 self-built microbial genome databases, including viruses, bacteria, fungi, and parasites, by BWA to generate the original mapping list. The self-built microbial genome reference database currently contains 6,039 bacteria, 2,700 DNA viruses, 1,064 fungi, 234 parasites, and 137 mycoplasma/chlamydia, all associated with human disease. Reference genomes in the database were downloaded from the National Center for Biotechnology Information (ftp://ftp.ncbi.nlm.nih.gov/genomes/). Count and normalize the number of uniquely aligned reads to obtain stringently mapped read numbers to species (Species_SMRN) and stringently mapped read numbers to genus (Genus_SMRN). CovRate and Depth were calculated for each microorganism using BEDTools[2]. Calculate the relative abundance of species (Species_Re_Abu), the relative abundance of genus (Genus_Re_Abu), the absolute abundance of species (Species_Abs_Abu), and the absolute abundance of genus (Genus_Abs_Abu). SHDI (Shannon's Diversity Index) for each microbial sequence map was calculated to explain the type of sequenced nucleic acid fragmentation or the tendency of fragment type to be evenly distributed in the overall sequence map. The highest SHDI is 1, an increase in SHDI indicates that the type of patch is increasing or that each type of patch is distributed in a balanced trend in the landscape, both with higher confidence in the microbial sequence alignment. For the software parameter flow of data preprocessing and database comparison, see Supplementary Method Table 1 and Supplementary Method Table 2.

**Metagenomic Data Release**

Due to the wide variability of DNA in samples, a simple cut-off threshold for the number of reads in an organism is not possible. This study is a verification of background bacteria, and no pathogen threshold interpretation rules were introduced. The exported data is divided into four data lists of bacteria, viruses, fungi, and parasites. Then the respective sorting criteria are based on species ranking, sequence number, and relative abundance. For the original data list of bacteria, see Supplementary Table 1. Because the amount of data fragments of viruses, fungi, and parasites are very small, the original data list is not displayed, only the diagram is shown, see Supplementary Fig. 1-6.

**Supplementary Method Table 1. Data preprocessing software and main parameters**

| Software-1 | get_umhost_IC_qc |
| --- | --- |
| Version No. | v 2.0 |
| Specific parameters | The parameters are fixed in the software, not optional |
| Main purpose | 1. Eliminate reads containing low-quality bases with a proportion greater than the threshold (average Phred quality score <20) 2. Eliminate reads containing N bases greater than the threshold (>10%) 3. Eliminate reads containing linker contamination 4. Eliminate low complexity reads |
| Software-2 | PRINSEQ-lite |
| Version No. | v 0.20.4 |
| Specific parameters | -derep -derep_min -lc_method -lc_threshold |
| Main purpose | Remove low-complexity sequences and repetitive sequences |
| Software-3 | samtools |
| Version No. | v 1.9 |
| Specific parameters | rmdup -s |
| Main purpose | Remove repetitive sequences |
